# The BCL-2 protein family: from discovery to drug development

**DOI:** 10.1038/s41418-025-01481-z

**Published:** 2025-04-09

**Authors:** Carlo M. Croce, David Vaux, Andreas Strasser, Joseph T. Opferman, Peter E. Czabotar, Stephen W. Fesik

**Affiliations:** 1 Department of Cancer Biology and Genetics and Comprehensive Cancer Center, The Ohio State University, Columbus, OH USA; 2 The Walter and Eliza Hall Institute, Parkville, VIC Australia; 3 Department of Medical Biology, University of Melbourne, Parkville, VIC Australia; 4 Department of Cell and Molecular Biology, St. Jude Children’s Research Hospital, Memphis, TN USA; 5 Department of Biochemistry, Pharmacology and Chemistry, Vanderbilt University, Nashville, TN USA

**Keywords:** Cancer, Drug development

## Abstract

The landmark discovery of the BCL-2 gene and then its function marked the identification of inhibition of apoptotic cell death as a crucial novel mechanism driving cancer development and launched the quest to discover the molecular control of apoptosis. This work culminated in the generation of specific inhibitors that are now in clinical use, saving and improving tens of thousands of lives annually. Here, some of the original players of this story, describe the sequence of critical discoveries. The t(14;18) chromosomal translocation, frequently observed in follicular lymphoma, allowed the identification and the cloning of a novel oncogene (*BCL-2*) juxtaposed to the immunoglobulin heavy chain gene locus (*IgH*). Of note, BCL-2 acted in a distinct manner as compared to then already known oncogenic proteins like ABL and c-MYC. BCL-2 did not promote cell proliferation but inhibited cell death, as originally shown in growth factor dependent haematopoietic progenitor cell lines (e.g., FDC-P1) and in *Eμ-Myc/Eμ-Bcl-2* double transgenic mice. Following a rapid expansion of the BCL-2 protein family, the Abbott Laboratories solved the first structure of BCL-XL and subsequently the BCL-XL/BAK peptide complex, opening the way to understanding the structures of other BCL-2 family members and, finally, to the generation of inhibitors of the different pro-survival BCL-2 proteins, thanks to the efforts of Servier/Norvartis, Genentech/WEHI, AbbVie, Amgen, Prelude and Gilead. Although the BCL-2 inhibitor Venetoclax is in clinical use and inhibitors of BCL-XL and MCL-1 are undergoing clinical trials, several questions remain on whether therapeutic windows can be achieved and what other agents should be used in combination with BH3 mimetics to achieve optimal therapeutic impact for cancer therapy. Finally, the control of the expression of BH3-only proteins and pro-survival BCL-2 family members needs to be better understood as this may identify novel targets for cancer therapy. This story is still not concluded!

## The cloning of the BCL2 gene

### Carlo M. Croce

In 1960 Peter Nowel and David Hungerford showed the presence of a small marker chromosome, the so-called Philadelphia chromosome, in more than 95% of chronic myeloid leukemias [1]. After the discovery of chromosome banding techniques by Torbjörn Caspersson, Lore Zech and their colleagues, it was possible to analyze the origin of the Philadelphia chromosome and in 1973 Janet Rowley showed that it derives from a reciprocal translocation between chromosomes 9 and 22 [2]. Since then, many different consistent chromosome translocations have been discovered in haematopoietic and solid tumors. In 1979 Manolov, Manolova, and Klein identified a t(8;14) translocation in most (~80%) Burkitt lymphomas [3]. The remaining ~20% of Burkitt lymphomas have the so called variant translocations, t(2;14) and t(8;22).

At that time, we were mapping human genes to their specific chromosome and found that the human immunoglobulin gene heavy chain cluster maps to chromosome 14 [4] and the human immunoglobulin lambda light chain locus maps on chromosome 22 [5], two chromosomes involved in translocations in Burkitt lymphoma. In my Nature paper of 1981, I suggested that the chromosome translocations in Burkitt lymphoma, a B cell malignancy, may involve the human immunoglobulin loci. At that time, although many consistent chromosomal translocations were observed in human malignancies, most of the scientific community believed that they were an epiphenomenon of the cancer process and not a cause. This belief vanquished in 1982 when my lab [6] and subsequently the Leder lab showed the juxtaposition of the human MYC oncogene, the homolog of the avian *MYC* gene responsible of lymphoma in chicken, to the human immunoglobulin heavy chain locus in Burkitt lymphoma cells. In a series of papers published in Science and PNAS in 1983 we also demonstrated that the *c-MYC* gene involved in the translocation is expressed at high level, while the *c-MYC* gene on the unaffected chromosome 8 is transcriptionally silent [7, 8]. In 1983 we also demonstrated that the t(2;8) and t(8;22) variant chromosomal translocations juxtaposed the *c-MYC* gene locus to either of the two immunoglobulin light chain loci (κ or *λ*). Since in Burkit Lymphoma the translocated *c-MYC* gene is transcribed at high level we speculated that the presence of enhancer elements in the human immunoglobulin loci are capable of activation of the *c-MYC* gene juxtaposed to the immunoglobulin heavy chain locus. Thus, the *c-MYC* gene involving chromosomal translocations were the first translocations resolved at the molecular level.

Uncovering the role of the *c-MYC* gene translocation in cancer, led us to speculate that chromosomal translocations involving the regions carrying immunoglobulin genes could be exploited to clone unknown oncogenes involved in the pathogenesis of human lymphomas and leukemias. Other translocations involving the human immunoglobulin heavy chain locus have been observed in several human lymphoblastoid malignancies [9–11].

In follicular lymphoma, the second most common lymphoma in humans, a chromosomal translocation was observed between human chromosome 14, where the immunoglobulin heavy chain locus resides, and chromosome 18 at band 18q21.3 [9, 11]. In the leukemic cell line 380, we identified chromosomal translocations at (8;14) and at (14;18) resulting in two 14q+ chromosomes, the t(14;18) being similar to that observed in follicular lymphomas, thus the DNA of 380 cells was digested with the restriction enzyme BAMH1 and analyzed by Southern blotting [12]. The result of this experiment showed that the 380 DNA contains two rearranged J_H_ segments of 18.5 and 14 kb. The same DNA fragments hybridized to the probe specific for the *C*_*μ*_ locus. Therefore we inferred that the two chromosomal rearrangements had occurred within the *J*_*H*_ segment of the Ig heavy chain gene locus. Since no expression of the Ig heavy chain locus was detected in the 380 cells, we concluded that the *C*_*μ*_ rearrangements were not productive.

**Fig. 1:**
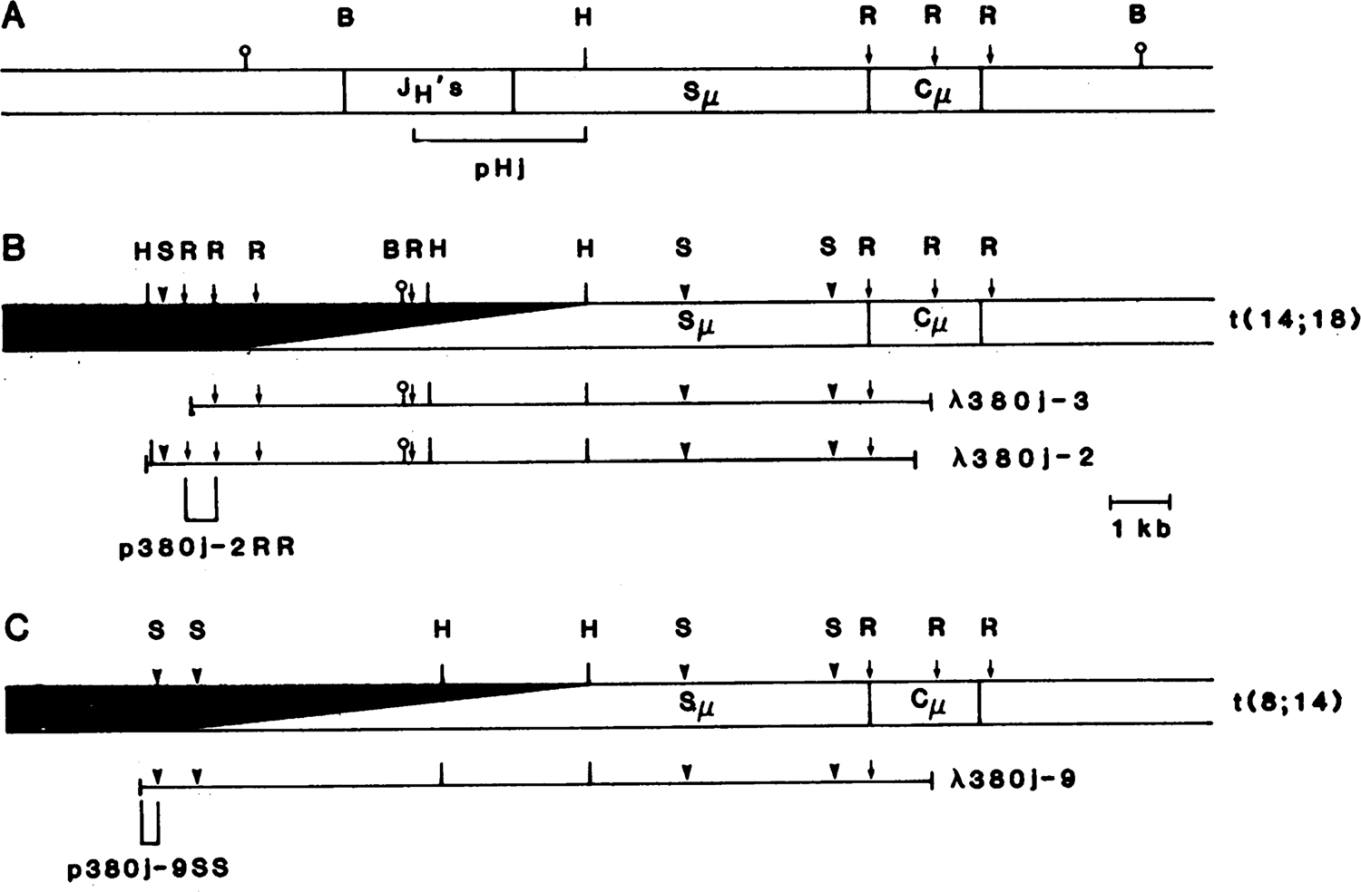
Chromosomal translocation mapping reveals the gene for BCL-2. Restriction maps of the germ line C1, gene (**A**) and of the two classes of recombinant clones from the l4q’ chromosomes resulting from the t(14;18) (**B**) and the t(8;14) (**C**) translocations. Abbreviations: H Hind III, R Eco RI, B Bam HI, and S Sst I. The black bars represent the chromosome 18-derived sequences in (**B**) and the chromosome 8-derived sequences in (**C**). The open bar represents the chromosome 14-derived sequences. (Reproduced from Ref. [12]).

At this point we prepared a genomic library from the DNA of the 380 cells that was partially digested with the restriction enzyme Sau3A and the two fragments of 14 and 23 kb in length were purified by sucrose gradient centrifugation [12] and then ligated to the DNA of the λ phage vector EMBL3A [12], which was cut with the enzyme BAMH1. After packaging in vitro ~42,000 recombinant phages were screened with a probe specific for the *J*_*H*_ DNA fragment. Nine recombinant clones were obtained, and restriction enzyme mapping allowed us to classify them into two groups representing fragments derived from the two 14q chromosomes [12]. In order to identify the group derived from chromosome 8 we subcloned DNA fragments 5’ of both clones *J*_*H*_ segments. These subclones were then used as probes in Southern blotting hybridization of DNA derived from rodent human hybrids containing either chromosome 14, or 8 or 18. We found that one probe, P380j-953, hybridized to the DNA of a hybrid containing only human chromosome 8. The same probe did not hybridize to hybrids containing only human chromosome 14. Therefore we concluded that the class of recombinants containing the p380j-955 segment carry the joining regions between chromosome 8 and 14 on the 14q32 chromosome (Fig. 1).

Since the clone shown in Fig. 1C is derived from the 14q+ chromosome of the t(8;14) translocation, clones λ380,-2 and λ380,-3 (Fig. 1B) should be derived from the 14q+ chromosome of the t(14;8) chromosome translocation. At this point, we hybridized the p38j-2 to rodent human hybrids containing either human chromosome 18 or 14. This probe hybridized to human DNA and to that from hybrids containing human chromosome 18 but not from hybrids containing human chromosome 14, indicating that clones λ380,-2 and λ380,-3 contain the joining between chromosome 14 and 18.

**Fig. 2:**
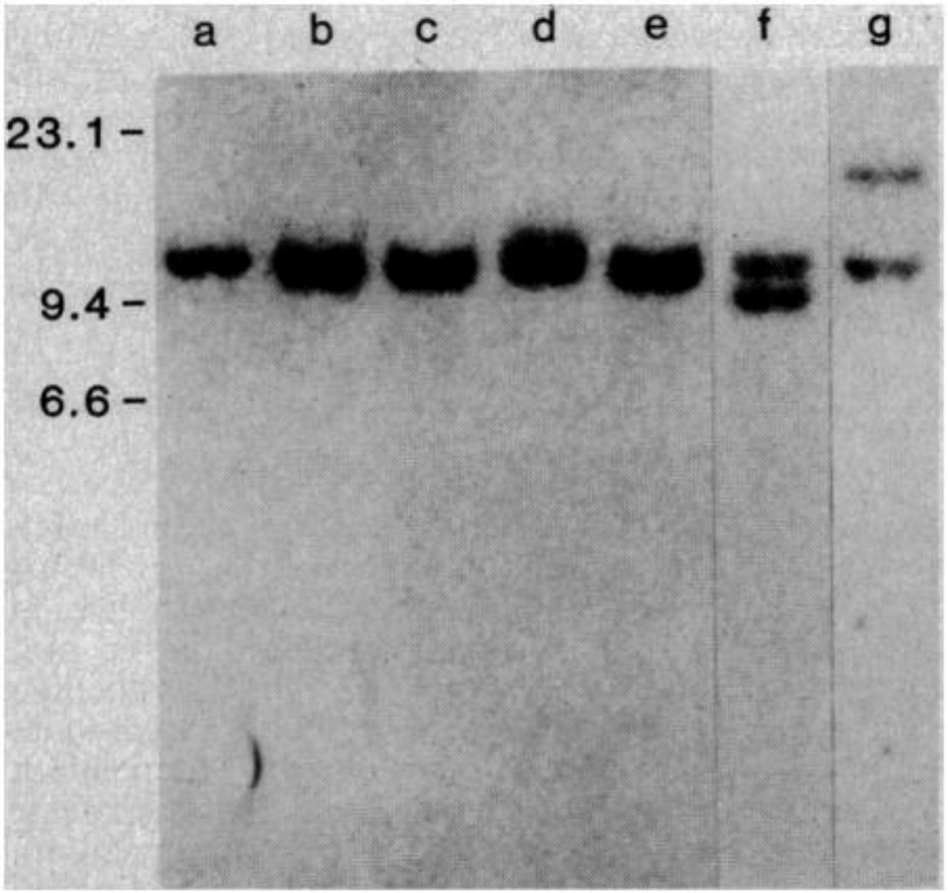
Southern blot hybridization with human chromosome 18-specific probe p380j-2RR of DNA from 380 leukemic cells and LN128 lymphoma cells. Human DNA’s were cut with Sst I and run on 0.7 percent agarose gel. The Southern blot filter was hybridized with 32P-labeled p380j-2RR and washed as described in the legend to Fig. 3. (Lane a) DNA from normal human peripheral blood lymphocytes; (lane b) DNA from human Tcell lymphoma cell line; (lane c) DNA from cells of CLL271 [a chronic lymphocytic leukemia with a t(1 1;14) chromosome translocation]; (lane d) DNA from Burkitt lymphoma cell line (Daudi) with t(8;14) translocation; (lane e) DNA from 545T T-cell line; (lane f) DNA from 380 leukemic cell line; (lane g) DNA from LN128 cells [human follicular lymphoma with a t(14;18) chromosome translocation]. (Reproduced from reference [12]).

At this point we asked the question whether the same chromosome 18 specific DNA segment cloned from 380 cells derived from a case of acute lymphocytic leukemia could detect rearrangements in follicular lymphomas with the same chromosomal translocation. The DNA from a patient with follicular lymphoma and a t(14;18) chromosomal translocation (LN128) showed both a germ line and a rearranged DNA fragment hybridizing to the p380j-2RR DNA probe (Fig. 2).

Thus the same DNA fragment derived from chromosome 18 is rearranged in both 380 leukemic cells and in LN128 follicular lymphocytic cells, suggesting that the same gene, we named *BCL2*, may be consistently involved in B cell malignancies with the t(14;18) chromosomal translocation. These results clearly indicated that immunoglobulin heavy chain DNA probes could be exploited to clone novel oncogenes juxtaposed to the heavy chain locus [12].

Two of the probes derived from chromosome 18 we obtained detected chromosomal rearrangements in ~60% cases of follicular lymphoma screened. Most of the rearrangements in band 18q21 were clustered in a very short stretch of DNA of ~21ϰb in lengths. Chromosome 18 specific DNA probes for areas flanking the rearrangement also detected RNA transcripts of 6ϰb in length in varying cell types. The gene encoding these transcripts (BCL2) seemed to be interrupted in most cases of follicular lymphoma.

We then carried out an analysis of the structures, transcripts, and protein products of *BCL2*. We found two BCL2 transcripts carrying two overlapping open reading frames, one of which is 717 nucleotides long and encodes a protein (BCL2 alpha) of 239 amino acid and a predicted molecular mass of ~26 K Daltons, while the other transcript encodes as protein of 205 amino acids (BCL2ß) with a predicted molecular mass of ~22 K Daltons that is identical to BCL2α except at the carboxyl terminus. The BCL2 protein produced in follicular lymphomas with or without *BCL2* gene rearrangements is identical to the BCL2 protein found in normal cells.

To summarize, in 1984 we cloned the human *BCL1* gene locus involved in mantle cell lymphoma, and then in 1984–1985 we cloned and characterized the human *BCL2* gene which is subject to the t14;18 chromosomal translocation found in most cases of follicular lymphoma, one of the most common human lymphomas [12, 13]. In 1986 we also reported the complete sequence of the *BCL2* mRNA and BCL2 protein [14]. In a series of papers in 1985–86 we also showed that the chromosomal translocations involved in these B cell malignancies occur as mistakes in the process of immunoglobulin gene rearrangement [15–17].

## Defining the function of BCL-2 and discovery of its relatives

### David Vaux, Andreas Strasser

The cytologist Janet Rowley showed that the Philadelphia chromosome seen in CML was the product of a 9;21 chromosome translocation [2] that later proved to involve the *ABL* proto-oncogene [18]. Moreover, Grace Shen-ong in Michael Cole’s lab showed that the *c-Myc* gene was involved in the t(12;15) translocations associated with plasmacytomas in mice [19].

So when Janet Rowley noticed the association of t(14;18) chromosomal translocations with follicular lymphoma [20], it was assumed that this might provide a path to identification of yet another oncogene.

The gene at the breakpoint on chromosome 18 was cloned and the cDNA sequenced by Yoshihide Tsujimoto in Carlo Croce’s laboratory [14], and by Mike Cleary in Jeffrey Sklar’s laboratory [21]. The thought was that the gene involved, designated *BCL-2*, would be an oncogene that promoted cell growth and proliferation, like *ABL* and *c-Myc*.

Jerry Adams wrote to Cleary in 1986 to ask for the *BCL-2* cDNA, hoping to make transgenic mice that overexpressed BCL-2, to see if they developed lymphoma. Cleary’s letter - that accompanied a tube with the *BCL-2* cDNA - said that he, Cleary, planned to make *BCL-2* transgenic mice. So, he asked that Jerry not do so. Jerry passed it to David Vaux and asked him to try to figure out if *BCL-2* was an oncogene.

At The Walter and Eliza Hall Institute (WEHI), Wendy Cook had shown that the viral onco-protein v-Abl was able to allow IL-3 dependent FDC-P1 mouse myeloid progenitor cells to proliferate in the absence of their requisite growth factor, and these cells, but not the parental line, made tumors in syngeneic mice [22].

Vaux thought that over-expression of BCL-2 in FDC-P1 cells might be able to do the same thing. To stably transfect the cells, he used a retroviral vector called *mpZen* [23] that had been designed by Iswar Hariharan, a PhD student with Jerry Adams who occupied the bench opposite his.

Initially Vaux was disappointed, because the BCL-2 expressing cells did not form colonies when cultured in the absence of IL-3, whereas, consistent with previous work, the v-Abl transfected cells formed nice colonies in soft agar under these conditions.

Hoping to find even the tiniest of colonies, he looked carefully at the cells, and although there were no colonies, the BCL-2 expressing FDC-P1 cells looked small, but otherwise healthy and refractile, even after many days culture without growth factor. In contrast, the control cells bearing an empty *mpZen* vector all appeared dark, shrunken, and dead.

To look further, Vaux plucked 18 independent FDC-P1 clones bearing the *mpZen-BCL-2* construct, and 18 control clones bearing the empty vector from the IL-3 containing soft agar cultures and transferred them into liquid culture. After expanding them in medium with IL-3, he thoroughly washed the 36 lines and split each into two: one set cultured in medium without IL-3, and the other in medium containing IL-3.

As expected, all the cells cultured with IL-3 proliferated exponentially regardless of the vector they had been transduced with. By day 4 in cultures devoid of IL-3, all the cells from the 18 clones with the empty vector had died. In contrast, almost all FDC-P1 clones bearing the *mpZen-BCL-2* vector contained many cells that looked healthy in the absence of IL-3. Importantly, when he restored IL-3 to these cultures, the BCL-2 expressing cells were able to proliferate once more, whereas the control cells did not.

This revealed that BCL-2 acted differently to oncogenic proteins such as ABL and c-MYC that promote cell proliferation. BCL-2 did not affect cell growth or proliferation, but it had a different property—it prevented the cells from killing themselves when they were deprived of their requisite growth factor [24]. The ability of BCL-2 to inhibit the death of cells deprived of growth factor was confirmed the following year by Timothy McDonnell in Stan Korsmeyer’s laboratory [25].

Vaux then used the *mpZen-BCL-2* virus to infect bone marrow cells from normal and *Eμ-Myc* transgenic mice, over-expressing c-MYC in B lymphoid cells. Only cells over-expressing both BCL-2 and c-MYC formed colonies in soft agar, and some eventually formed lymphomas when transplanted into mice. This indicated that BCL-2 and c-MYC may synergize to transform B lymphoid cells, but he held a lingering worry that if the *Eμ-Myc* transgenic mice that provided the bone marrow had a pre-clinical lymphoma, the results might have been skewed. He therefore talked to Jerry and asked if they could call Mike Cleary to seek his permission to make *Eμ-Bcl-2* transgenic mice to cross them with the *Eμ-Myc* transgenic mice. Mike agreed, and with help from ML “Sue” Bath, Vaux set about generating *Eμ-Bcl-2* transgenic mice.

Vaux and Bath produced over two dozen primary lines, and with Alan Harris’s help, and after testing their expression of *BCL-2* by Northern blot analysis, they crossed one of the most promising lines with the *Eμ-Myc* transgenic mice. By this time, Tim McDonnell in Stan Korsmeyer’s laboratory had beaten them to make *Eμ-Bcl-2* transgenic mice [25], but shortly before Vaux headed off to Stanford, the first three *Eμ-Myc/Eμ-Bcl-2* double transgenic mice had developed lymphoma at only a few weeks of age, confirming the oncogenic synergy between these two oncogenes. Andreas Strasser joined Suzanne Cory’s laboratory at WEHI and conducted a thorough characterization of several of the *Eμ-Bcl-2* transgenic lines and the development of lymphoma in the *Eμ-Myc/Eμ-Bcl-2* double transgenic mice [26, 27].

Andreas with Suzanne Cory and Alan Harris found that *Eμ-Bcl-2* transgenic mice only had a low incidence of lymphoma (5–10% in the first year of life) [28]. Together with the finding of potent acceleration of c-MYC driven lymphoma development by BCL-2 over-expression (see above; [26]), this indicated that inhibiting cell death promoted tumorigenesis by keeping alive cells sustaining stress from oncogene activation, thereby facilitating subsequent steps in neoplastic transformation, including enabling the acquisition of further oncogenic mutations. Gerard Evan’s and Doug Green’s groups confirmed this synergy of c-MYC and BCL-2 by showing that deregulated expression of c-MYC caused apoptosis that could be blocked by BCL-2 [29–31].

Unexpectedly, even though the incidence of lymphoma was low in *Eμ-Bcl-2* transgenic mice, these animals developed with high incidence fatal autoimmune disease, resembling human systemic lupus erythematosus (SLE) [32]. This demonstrated for the first time that defects in apoptosis can cause autoimmune disease. Subsequent work from Andreas, Philippe Bouillet, Anselm Enders, David Tarlinton, Suzanne Cory and Jerry Adams revealed that BCL-2 over-expression or loss of one of its inhibitors, BIM (see below), could prevent the killing of autoreactive T cells and B cells during their development in the thymus [33, 34] or bone marrow [35], respectively. This identified the molecular mechanism of clonal deletion for self-tolerance in the immune system enunciated by Sir MacFarlane Burnet [36].

Meanwhile, now at Stanford, David Vaux wanted to perform a zoo blot so he could use low stringency Southern blotting to find *BCL-2* homologs in other organisms [37]. When he asked Stuart Kim for some *C. elegans* DNA, Stuart told him he had learnt the technique to generate transgenic worms. No one had ever made a transgenic worm that expressed a mammalian gene, but together they hatched a plan to take a long-shot and produce transgenic *C. elegans* that expressed human BCL-2, to see if it would affect the death of the 131 cells that are programmed to die during development of this worm [38].

Vaux inserted the human *BCL-2* cDNA into a heat-shock inducible vector kindly provided by Peter Candido [39]. Stuart injected the construct, along with a *Rol6* marker, into *CED-1* mutant worms [40] (defective in removal of dead cells), so that it would be easier to count the cell corpses. They found that when human BCL-2 was expressed, the number of corpses decreased by about 65%, indicating that human BCL-2 protein could interfere with the cell death machinery in *C. elegans*. This meant that the mechanism for apoptosis in mammalian cells was closely related to the mechanism that caused programmed cell death in the worm [41]. In 1994, Michael Hengartner and Bob Horvitz reported the sequence of the worm cell death inhibitory gene *ced-9* and found that the gene with closest similarity was *BCL-2* [42].

The realization of the conservation of programmed cell death between humans and worms kickstarted genetic screens and biochemical experiments that led to the discovery of the currently well-known regulators of apoptosis, including other anti-apoptotic BCL-2 proteins, their pro-apoptotic relatives (i.e., the BH3-only proteins and the BAX/BAK/BOK effectors of apoptosis), the aspartate specific cysteinyl proteases (caspases) and their adapters. However, there are notable differences in the control of apoptosis between *C. elegans* and mammals. In the worm, CED-9 appears to inhibit programmed cell death by preventing the adapter CED-4 from activating the caspase, CED-3 [43], and caspase activation constitutes the point of no return in cell death signaling in this organism. While for some time it was assumed that this would also hold true for mammalian cells, this view was overturned by the demonstration that the loss of the critical initiator caspase, caspase-9, and its adapter, APAF-1, did not cause any increase (i.e., abnormal survival) of lymphoid cells in mice, whereas over-expression of BCL-2 or loss of its pro-apoptotic relatives BIM or BAX plus BAK, caused a ~ 5-fold increase in lymphoid cells [44–47]. This and elegant biochemical work (reviewed in [48]) demonstrated that BCL-2 and its anti-apoptotic relatives do not directly regulate caspase activation but do so by preventing mitochondrial outer membrane permeabilization (MOMP) by restraining BAX and BAK, the effectors of apoptosis in mammalian cells that have no counterparts in *C. elegans*. Substantial MOMP appears to constitute the point of no return in apoptosis signaling in mammals, although recent work has shown that at least some cell types can recover from limited MOMP, continuing to survive while activating diverse cellular stress responses [49].

Many years of biochemical, molecular biology and structure biology work from many laboratories (see accompanying sections by *P Czabotar and S Fesik*) led to a solid understanding of apoptosis signaling in mammalian cells. BAX and BAK are the essential effectors of apoptosis that when unleashed cause MOMP [48, 50]. In healthy cells BAX and BAK are restrained by direct binding by the pro-survival BCL-2 proteins (BCL-2, BCL-XL, MCL-1, BCL-W, A1/BFL1). In response to diverse stresses, the levels of the BH3-only proteins (BIM, PUMA, NOXA, BID, BMF, BAD, BIK, HRK), that are critical for the initiation of apoptosis, are increased through complex (for some of these proteins still not fully understood) transcriptional and post-transcriptional processes. The BH3-only proteins can bind with very high affinity to the pro-survival BCL-2 proteins, thereby unleashing the effectors of apoptosis, BAX and BAK. Some BH3-only proteins have been reported to be able to also directly activate BAX and BAK [51]. However, elegant studies in which all BH3-only proteins had been genetically removed from cell lines provided evidence that this action of BH3-only proteins is not essential for BAX/BAK activation and cell killing [52]. Collectively, these discoveries have inspired and focused translational work; i.e., to prevent aberrant cell death in degenerative diseases we need inhibitors of BAX and BAK, not inhibitors of caspases, whereas the killing of unwanted cells, e.g., cancer cells, is best achieved by compounds that mimic the function of the pro-apoptotic BH3-only proteins [see chapter by *Steve Fesik*].

**Fig. 3:**
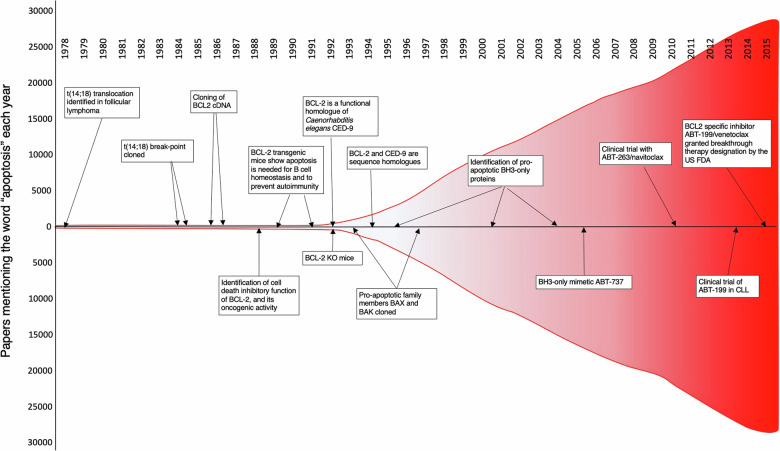
Explosive growth of research on apoptosis. The numbers of papers mentioning the term apoptosis each year are graphed in red, and some of the key discoveries that propelled this growth are mentioned in the boxes. Although the term apoptosis was adopted for cell death in 1972, there was little interest until BCL-2 was shown to inhibit cell death, the first component of the mechanism to be recognised.

However, despite the advancement of the field (Fig. 3), important questions remain. For example, (1) how are the levels of the pro-apoptotic BH3-only proteins controlled and can we manipulate these processes for cancer therapy (e.g., see recent work on STING pathway signaling [53], (2) how can we make inhibitors of BCL-XL and MCL-1 safe and effective for cancer therapy, (3) what are the relative importance of the well-known anti-apoptotic functions of MCL-1 and BCL-XL vs their still less well defined apoptosis unrelated functions (e.g., see [54, 55]) and can we harness this knowledge to develop new agents for cancer therapy, (4) what are the processes that interconnect apoptosis signaling with other forms of programmed cell death more directly than previously anticipated (e.g., see [56, 57]) and could understanding of these processes lead to novel cancer therapies, for example by combining BH3 mimetic drugs to trigger apoptosis with inducers of ferroptosis, necroptosis or pyroptosis [58] and, conversely, do we need to simultaneously inhibit multiple programmed cell death pathways and/or the processes that connect them to treat degenerative diseases? Hopefully, 20 or 40 years from now *Cell Death and Differentiation* will publish a reminiscence on currently imminent discoveries answering these questions that will hopefully have considerable therapeutic impact.

## Expanded BCL2 family reveals distinct, non-redundant roles in biology

### Joseph T. Opferman

Forty years ago, the discovery of BCL-2, an anti-apoptotic protein, marked a breakthrough in our understanding of the regulation of programmed cell death [12, 14]. Researchers found that a translocation in follicular lymphoma involving chromosomes 14 and 18 positioned BCL-2 near a high-expression region [4, 7, 59], causing overproduction of lymphoma cells [15]. Unlike typical oncogenes that drive cell proliferation, these scientists found that BCL-2 prevented cell death; its anti-apoptotic function allowed cancer cells to survive despite violating cellular checkpoints. After BCL-2’s initial discovery, scientists worldwide identified additional BCL-2 family members, revealing a complex network that regulates apoptosis [24]. By the 1990s, it was recognized that pro-apoptotic and anti-apoptotic relatives within the family share so-called BCL-2 homology (BH) domains [35]. Key proteins identified include BAX and BAK, which promote apoptosis through the release of mitochondrial proteins such as cytochrome *c*, and BCL-XL and MCL-1, which help cells resist death. Furthermore, multiple BH3-only proteins were identified as sensors of cellular stress. Together, these proteins function as a “rheostat” for cell death, controlling the decision between survival and death in response to cellular signaling [37].

**Fig. 4:**
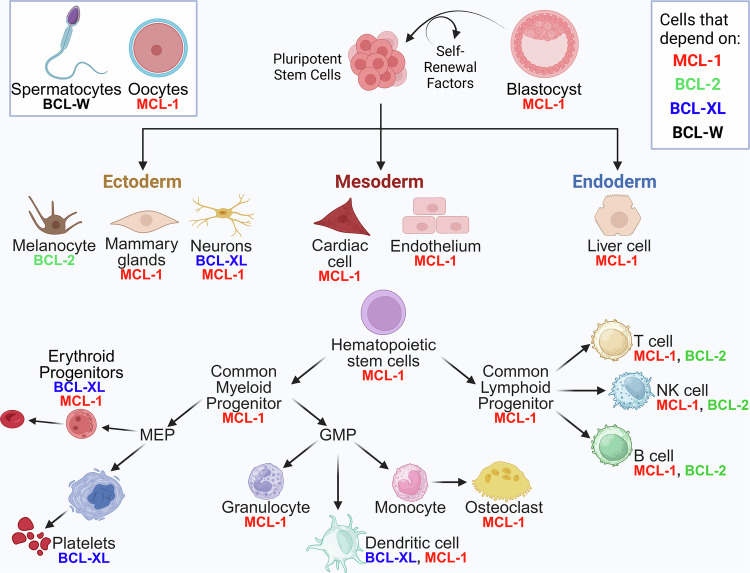
Requirements for expanded BCL-2 family members in mammalian cellular homeostasis. Genetic ablation studies have revealed that the various anti-apoptotic BCL-2 family members play selective, critical roles in promoting the survival of many cell types. The requirement for indicated pro-survival family members is indicated by each cellular lineage [60].

Studies using knockout mice—genetically engineered to lack specific BCL-2 family genes—have been instrumental in understanding the requirements of individual BCL-2 family members in normal development and cellular function [60]. These studies have revealed that various BCL-2 family proteins play distinct, non-redundant roles in tissue maintenance and organismal survival. For example, BCL-2-knockout mice showed early developmental defects, particularly in lymphoid organs and melanocytes, due to excessive apoptosis, underscoring BCL-2’s essential role in the survival of specific cell lineages [61, 62]. In contrast, BAX and BAK double-knockout mice displayed severe developmental issues and died shortly after birth; they exhibited a dramatic accumulation of lymphocytes, revealing that these pro-apoptotic proteins are crucial for normal cell turnover [50]. Interestingly, mice lacking anti-apoptotic members like BCL-XL do not survive to birth due to massive cell death in the developing nervous and maturing erythrocytes [63], and tissue-specific genetic ablation and inhibitor studies revealed that BCL-XL plays an important role in promoting the survival of mature platelet cells [64, 65]. Likewise, the germline knockout of MCL-1 also results in early embryonic lethality of the developing blastocyst [66]. Tissue-specific genetic deletion of MCL-1 revealed that it plays essential roles in promoting the survival of many cell lines including most hematopoietic lineages, neurons, and adult cardiomyocytes [67, 68]. The study of various knockout mouse models has highlighted that each anti-apoptotic BCL-2 family member has specific, context-dependent roles crucial for normal physiology [60] (Fig. 4). This knowledge has been invaluable in guiding therapeutic strategies targeting BCL2 proteins, helping researchers anticipate potential side effects and refine approaches to selectively inhibit these proteins in cancer cells without disrupting essential functions in healthy cells.

Beyond their roles in the regulation of apoptosis regulation, BCL-2 family members are involved in a variety of non-apoptotic cellular processes essential for maintaining cellular health and functionality. Research has shown that these proteins play critical roles in mitochondrial dynamics, including processes like mitochondrial fission and fusion, which are crucial for cellular energy balance and the response to metabolic stress. For example, MCL-1 and BCL-XL interact with mitochondrial proteins to regulate energy production, influencing ATP synthesis and reducing oxidative stress [69, 70]. Additionally, BCL-2 family proteins are involved in calcium homeostasis by controlling calcium release from the endoplasmic reticulum [71, 72]. BCL-2 family members also contribute to autophagy regulation; BCL-2 and MCL-1, for instance, inhibit autophagy, balancing cell survival during nutrient scarcity [73]. These non-apoptotic functions of BCL-2 proteins have broad implications for cell physiology and are critical in many diseases beyond cancer, including neurodegeneration, cardiovascular disorders, and immune system dysregulation. This expanding view of BCL-2 family proteins highlights their multifaceted roles in cell biology and points to new therapeutic opportunities for targeting these proteins in a range of diseases where apoptosis-independent functions are disrupted [58].

The development of small molecules that inhibit BCL-2 family proteins marked a breakthrough in cancer treatment by promoting apoptosis in cancer cells, see section by Fesik. Many cancers exploit anti-apoptotic BCL-2 proteins—like BCL-2, BCL-XL, and MCL-1—to evade cell death. By the early 2000s, researchers had developed “BH3 mimetics,” compounds that block these anti-apoptotic proteins and tip the balance toward cell death. Venetoclax, the first FDA-approved BH3 mimetic, specifically targets BCL-2 and has shown efficacy in treating chronic lymphocytic leukemia and other hematological malignancies [74]. Current research suggests that small molecule antagonists targeting BCL-XL and MCL-1 could improve outcomes for cancers resistant to BCL-2 inhibition. Despite the success of venetoclax, creating effective inhibitors for other BCL-2 family members has remained challenging due to their essential roles in healthy cells. Emerging strategies like PROTACs, which degrade target proteins, and combination therapies may help minimize side effects, offering new, personalized treatments for cancers relying on these proteins.

Future BCL-2 research promises new insights into the cell biology of and therapies for diverse diseases. Studies are still uncovering BCL-2 proteins’ non-apoptotic functions in metabolism, mitochondrial dynamics, and immunity, which may reveal their roles in neurodegeneration, autoimmune, and cardiovascular disorders. Efforts are currently underway to create next-generation, highly selective inhibitors with fewer off-target effects to enhance personalized cancer treatments, especially for resistant types. Advances in structural biology are refining drug design, while combination therapies with BCL-2 inhibitors and other treatments have the potential to boost treatment efficacy – this research will continue to lead to groundbreaking therapies, benefiting a broad spectrum of patients with various medical conditions.

## Structural characteristics of the BCL-2 family

### Peter Czabotar

**Fig. 5:**
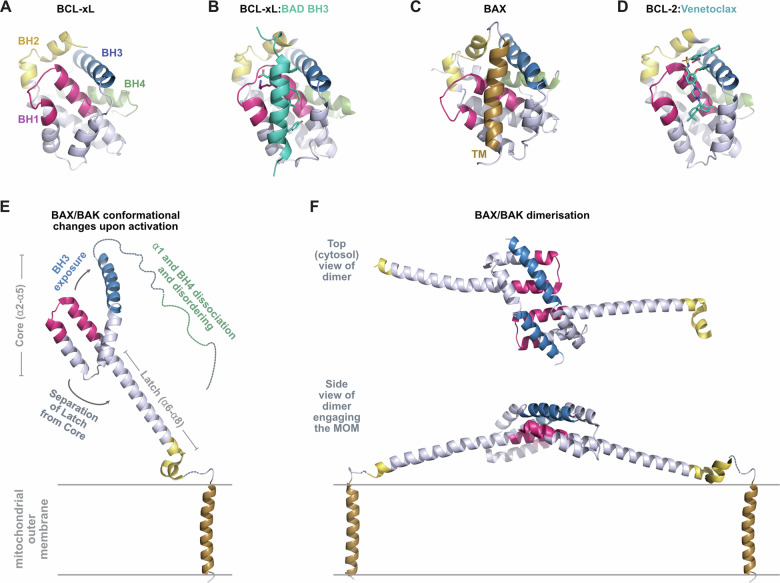
Structural motif of the BCL-2 family members. **A** The structure of BCL-XL (PDB code 1MAZ [75]) revealed an 8-helix conformation that has become known as the BCL-2 fold. Regions containing BCL-2 homology (BH) domains are colored and labeled. **B** The structure of the BCL-XL:BAD complex (PDB code 1G5J [76]). This and other structures revealed the details of the interaction interface between BH3 domains (cyan) and the hydrophobic groove of pro-survival proteins. **C** The full length structure of the pro-apoptotic effector protein BAX (PDB code 1F16 [78]) prior to activation. **D** Structure of BCL-2 in complex with Venetoclax (cyan in stick format, PDB code 6O0k [146]). **E** Key structural conformational changes that BAX and BAK undergo upon activation (here shown for BAK from PDB codes 7K02 [147] and 7OFO [148]). **F** Activated BAX and BAK form dimers via reciprocal insertion of their BH3 domains into a neighboring groove (here BAK, PDB code 7K02 [147]). These dimers serve as foundational units for the formation of higher order oligomers that permeabilise the mitochondrial outer membrane.

In 1996 the team at Abbott Laboratories, led by Stephen Fesik, solved the first structure of a BCL-2 family member (for BCL-XL [75]), revealing a novel fold that has since proved to be a defining characteristic for structured members of the family. Consequently, the moniker BCL-2 not only applies to this family of proteins but is also used to describe the structure that they adopt. The BCL-2 fold consists of a bundle of alpha helices surrounding a predominantly hydrophobic central helix (referred to as α5) (Fig. 5A), an arrangement that creates a hydrophobic groove on one surface of the protein that subsequently proved to be the major site for interaction with other family members [76, 77] (Fig. 5B). The groove is in part formed by three of the BCL-2 homology (BH) domains, consecutively by sequence the BH3, BH1 and BH2 domains. Structured family members include those with pro-survival function, e.g., BCL-2, BCL-XL, MCL-1, and viral pro-survival homologs, the pro-apoptotic effectors BAX, BAK, and BOK, and the atypical BH3-only family member BID. Many structures of BCL-2 family members have now been solved, both with and without bound BH3s from partner molecules (see below). The majority of these were solved using constructs for which the hydrophobic α9 transmembrane (TM) domain had been removed to enable protein expression. This region of the protein is predicted to be embedded in the outer mitochondrial membrane when located at this surface and is thus not expected to significantly influence the BCL-2 fold in this setting. However, some family members, notably BAX, have the capacity to shuttle between a soluble form in the cytosol and a membrane bound form at the mitochondria. The structure of full-length BAX from Nico Tjandra’s group [78], subsequently revealed how this can occur, with the hydrophobic TM domain able to insert into the surface hydrophobic groove (Fig. 5C), revealing a mechanism by which family members can exist in both soluble (TM in groove) and membrane bound (TM in lipid bilayer) states.

Members of the remaining BCL-2 family group, the BH3-only proteins, are in general intrinsically disordered when not bound to a partner [79]. Their BH3 domains form an alpha helix when interacting with the hydrophobic groove of a pro-survival family member (Fig. 5B), producing an amphipathic entity characterized by a series of hydrophobic residues on one surface, and a conserved aspartate on the other (residues shown in stick format on Fig. 5B). These residues form important interactions with partner proteins, with the hydrophobic residues projecting into hydrophobic pockets within the hydrophobic groove, and the aspartate forming a salt bridge with a conserved arginine residue within the BH1 domain on the periphery of the groove. BH3-mimetic drugs, such as Venetoclax, are designed to imitate these interactions (Fig. 5D), thereby competing for binding to pro-apoptotic partners at this interface and thus initializing apoptosis in cancer cells, as discussed elsewhere in this article.

As mentioned above, the BH3-only protein BID is unusual for its sub-group in that prior to activation by proteolysis it also adopts the BCL-2 fold and thus resembles the pro-survival proteins and the effectors BAX and BAK [80, 81]. BID also contains both a BH3 and BH4 domain [82], but no BH1 or BH2. Upon cleavage in the loop region N-terminal to the BH3 domain [83], and in the presence of a membrane [84], BID unfolds to reveal its BH3 domain which can then interact with partner family members via the same set of interactions as other BH3-only proteins. However, to add to the intrigue of BID, recent evidence indicates that in certain settings it may be able to act like BAX and BAK and permeabilise membranes to initiate apoptosis [85, 86]. These unusual characteristics suggest that BID does not belong to the BH3-only group of the BCL-2 family but instead should be placed in a category of its own.

It is surprising that members of a family of proteins can adopt the same fold but have opposing biological function, i.e., either pro-survival as in the case of BCL-2, or pro-apoptotic as in the case of BAX and BAK. It turns out that this is capacity is related to differences in their response to interaction with other family members. For the pro-survival group, interaction of the BCL-2 fold with a partner’s BH3 domain leads to a stable complex with a tight affinity. In contrast, the fold is meta-stable for effector family members such as BAX and BAK. For these proteins, interaction with a partner’s BH3 domain, either at the hydrophobic groove [87–89], and/or potentially at a second site on the opposing face [90], leads to activation and a structural reconfiguration. These conformational changes include dissociation and disordering of the region N-terminal to the α2, exposure of the BH3 located within the α2, and separation of a “core” region (α2–α5) and latch region (α6–α8) (Fig. 5E). BOK is also typically classed as a pro-apoptotic effector family member and, like BAX and BAK, adopts a version of the BCL-2 fold that can undergo conformational change [91, 92]. However, BOK does not require activation to initiate unfolding, indicating that it is more intrinsically unstable than either BAX or BAK. Instead, BOK activity appears to be regulated by protein turn over through targeting to the proteasomal degradation [93]. It is unclear what differentiating sequence and/or structural features lead to the distinct stability properties of pro-survival versus pro-apoptotic effector proteins. However, functionally, it is an important defining characteristic between these groups, and one that is required for the membrane permeabilizing activity of the latter group.

One of the conformational changes that occurs upon activation of BAX and BAK, exposure of the embedded BH3 domain, had been postulated from earlier biochemical studies [89, 94, 95], and the observation that this region interacted with the hydrophobic groove of pro-survival proteins in an inhibited complex [96–98]. A second change, dissociation of the α1 helix, had also been surmised from earlier experiments, notably from observations that this region on activated BAX was recognized by conformation specific antibodies [99, 100]. Additionally, we identified the third conformational change mentioned above, separation of a “latch” region (α6 to α8) from a “core” region (α2 to α5) [87, 101]. In the absence of a neutralizing pro-survival protein to bind to the exposed BH3 domain, these core domains form into symmetric homodimers, mediated by the insertion of the exposed BH3 domain into the groove of a likewise activated and conformationally altered neighbor [87, 94, 101, 102] (Fig. 5F). These dimers are amphipathic, with the hydrophobic surface (bottom of structure on side view in Fig. 5F), predicted to engage the mitochondrial outer membrane. The dimers serve as building blocks for the formation of higher order oligomers, although exactly how these are coordinated is not yet settled. Our studies suggest oligomerisation via lipid mediated interactions [103], studies from the Kluck group propose clustering through membrane forces [104]. Nonetheless, these complexes grow in size and shape on the mitochondrial outer membrane [105, 106] to form pores that release factors from inside, initially proteins such as Cytochrome c to initiate apoptosis [107, 108], or if left unchecked, very large structures such as mtDNA to initiate cGAS/STING signaling [109–112]. Within these pores the core regions of the dimers are believed to line the pore lumen [113, 114], although the topology of these structures is yet to be resolved (see [115] for further discussion).

Significant progress has been made in understanding how the structural characteristics of the BCL-2 family dictate their function since the first description of the BCL-2 family fold in 1996. However, we still fundamentally do not understand at the molecular level how oligomers of BAX, BAK and BOK are able to mediate the critical step of mitochondrial outer membrane permeabilisation for apoptotic signaling. Understanding how these proteins initiate membrane permeabilisation, line and maintain the pore lumen, and how drugs can be developed to manipulate their activation and pore forming capacity, will be the goal of structural, biophysical and drug discovery efforts within the field in years to come.

## Discovery and development of Bcl-2 family inhibitors

### Stephen W. Fesik

**Fig. 6:**
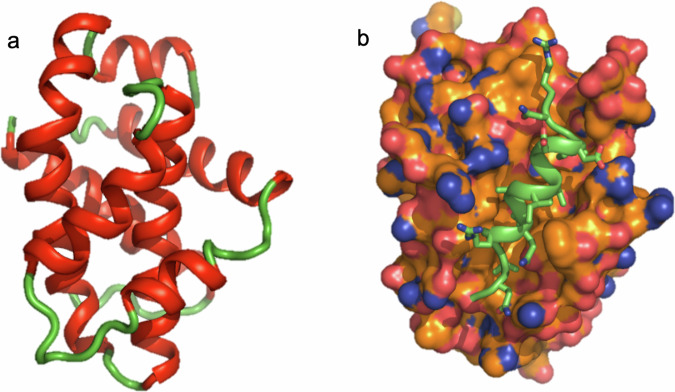
The three-dimensional structure of BCL-XL. The structure of **a** BCL-XL alone and **b** when complexed to the BAK peptide.

The BCL-2 family of proteins regulate apoptosis (programmed cell death)—an evolutionary conserved process for removing aged, damaged, or unnecessary cells [116]. The family is composed of proapoptotic (prodeath) proteins such as BAK, BAX, BAD, BID, NOXA, and others as well as antiapoptotic (prosurvival) proteins such as BCL-2, BCL-XL, BCL-W, MCL-1, and BFL-1 [117]. A hallmark of cancer is to evade programmed cell death [118]. One way that this is accomplished is to upregulate the antiapoptotic BCL-2 family proteins, which can bind to proapoptotic BCL-2 proteins and neutralize them [119]. Thus, a small molecule that binds to a prosurvival BCL-2 protein can prevent its interaction with a prodeath protein and should kill a cell. In principle, the antiapoptotic members of the BCL-2 family would be excellent targets for the treatment of cancer, providing that the small molecule could achieve a therapeutic window and kill the cancer while sparing normal cells. The small molecule must also be able to block a protein-protein interaction which was thought to be a very challenging task when we started this project 30 years ago [120]. To examine this possibility, we determined the first structure of a BCL-2 family protein, BCL-XL (Fig. 6a) [75], and subsequently, the structure of BCL-XL complexed to a peptide derived from the proapoptotic protein BAK (Fig. 6b) [77]. The structures revealed a hydrophobic groove in BCL-XL which interacts with an amphipathic helix of BH3 region of BAK. Based on the structure, we hypothesized that BCL-XL and other antiapoptotic members of the BCL-2 family might be druggable.

**Fig. 7:**
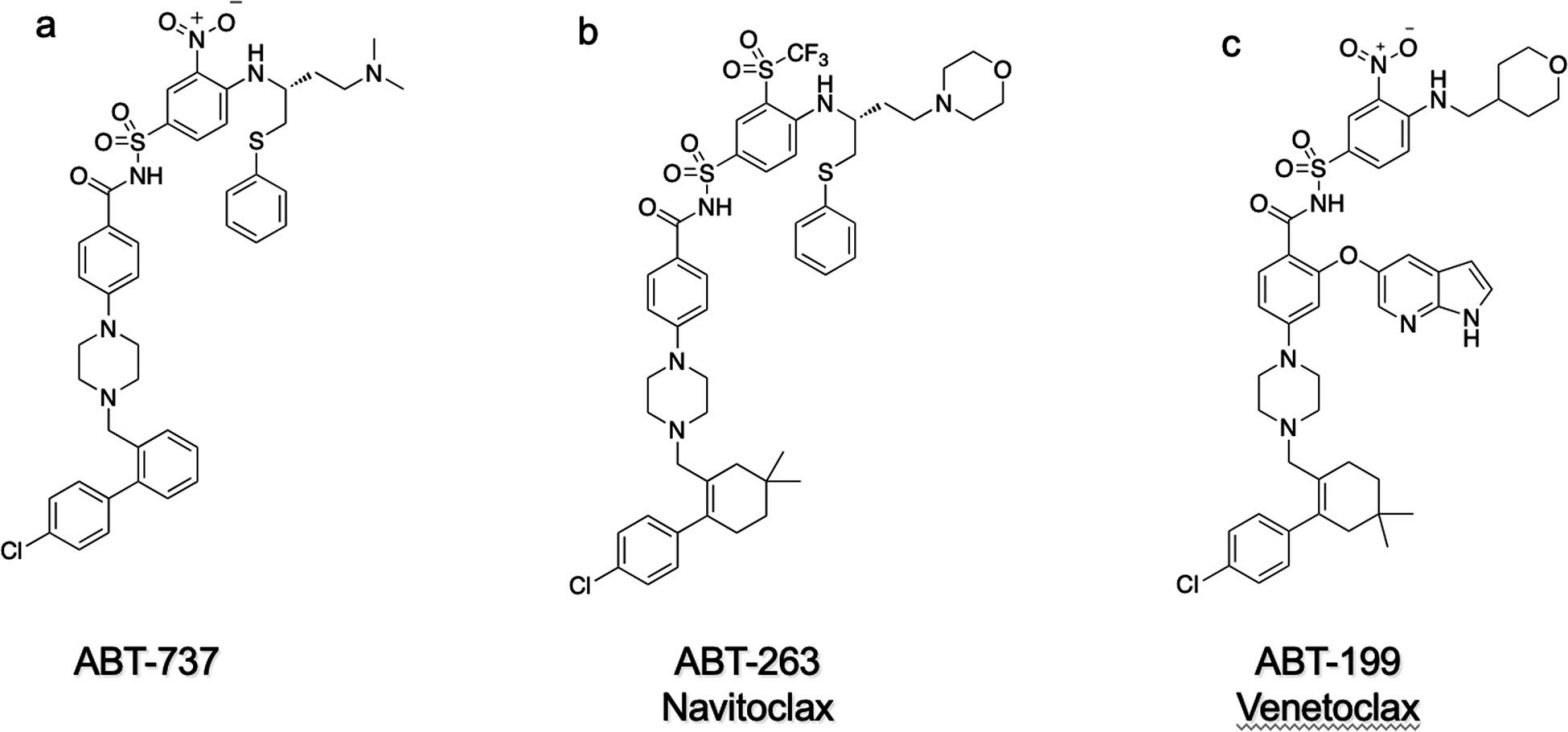
The structures of BCL-2 family inhibitors. **a** ABT-737 [123], **b** ABT-263 (navitoclax) [124], and **c** ABT-199 (venetoclax) [74].

To test this hypothesis, we conducted a high throughput screen of the Abbott (Abbvie) library, but did not find any good hits. In contrast, we did identify hits using a fragment-based method that we developed for discovering high affinity ligands for proteins which we called SAR by NMR because Structure Activity Relationships are obtained from NMR [121]. Using this fragment-based method, small organic molecules (fragments) that bind to proximal subsites of a protein are identified by NMR and linked together to produce high affinity ligands as demonstrated for the FK506 binding protein [121] and the matrix metalloproteinase, stromelysin [122]. When applied to BCL-XL we identified 4’-fluoro-biphenyl-4-carboxylic acid and 5,6,7,8-tetrahydro-napthelen-1-ol that bound to proximal sites in the hydrophobic groove of BCL-XL [123]. Analogs of these hits were linked together, and the linked molecules were optimized using structure-based design and extensive medicinal chemistry to produce the first potent BH3 mimetic, ABT-737 (Fig. 7a), that inhibited the antiapoptotic proteins BCL-XL, BCL-2, and BCL-W [123]. This was one of the first examples of successfully targeting a protein-protein interaction and showed that the antiapoptotic Bcl-2 proteins were indeed druggable. However, ABT-737 was not orally bioavailable which would limit chronic single agent therapy and flexibility in dosing when combined with other agents. Fortunately, an analog of ABT-737, ABT-263 (Fig. 7b), was discovered that potently inhibited the same prosurvival proteins, was orally bioavailable, and exhibited complete tumor regressions in xenograft tumor models [124]. ABT-263 (navitoclax) entered clinical trials as a single agent for the treatment of lymphoid malignancies. The dose limiting toxicity observed was thrombocytopenia which was found to be due to inhibition of BCL-XL [64, 65]. This prompted the discovery of a BCL-2 selective inhibitor, ABT-199, which was achieved by determining the x-ray structure of BCL-2 complexed with an analog of ABT-263 that lacked the S-phenyl group [74]. In the structure, a tryptophan from an adjacent BCL-2 protein occupied the same space as the S-phenyl of ABT-263 which was ultimately mimicked by an azaindole analog of ABT-263 to produce ABT-199 that exhibited 3 orders of magnitude less binding to BCL-XL and did not cause a reduction in platelets. Based on its remarkable efficacy and lack of significant toxicities [74], ABT-199 (Venetoclax) was approved for patients with chronic lymphocytic leukemia [125] and subsequently AML [126].

In order to treat solid tumors, inhibition of BCL-XL was important since overexpression of BCL-XL is commonly observed in solid tumors. Therefore, navitoclax was combined with docetaxel and other chemotherapeutic agents and tested for treating solid tumors in the clinic. In addition to the expected thrombocytopenia (which was manageable) due to the inhibition of BCL-XL, dose limiting neutropenia was observed which was believed to be caused by the inhibition of BCL-2 coupled with the effects caused by chemotherapy [127]. Thus, a BCL-XL selective inhibitor was sought that might alleviate neutropenia. The discovery of potent, orally bioavailable selective inhibitors of BCL-XL was achieved using high throughput screening combined with fragment-based methods and structure-based design [128–130]. However, these inhibitors caused rapid and severe cardiotoxicity, which stopped their further development. To selectively inhibit BCL-XL in solid tumors but not in the heart, a strategy was proposed using an antibody-drug conjugate in which a BCL-XL warhead was linked to a tumor targeting antibody. After extensive optimization of the warhead, linker, and antibody, the first selective BCL-XL-targeting agent was discovered [131] which recently entered clinical trials [132].

**Fig. 8:**
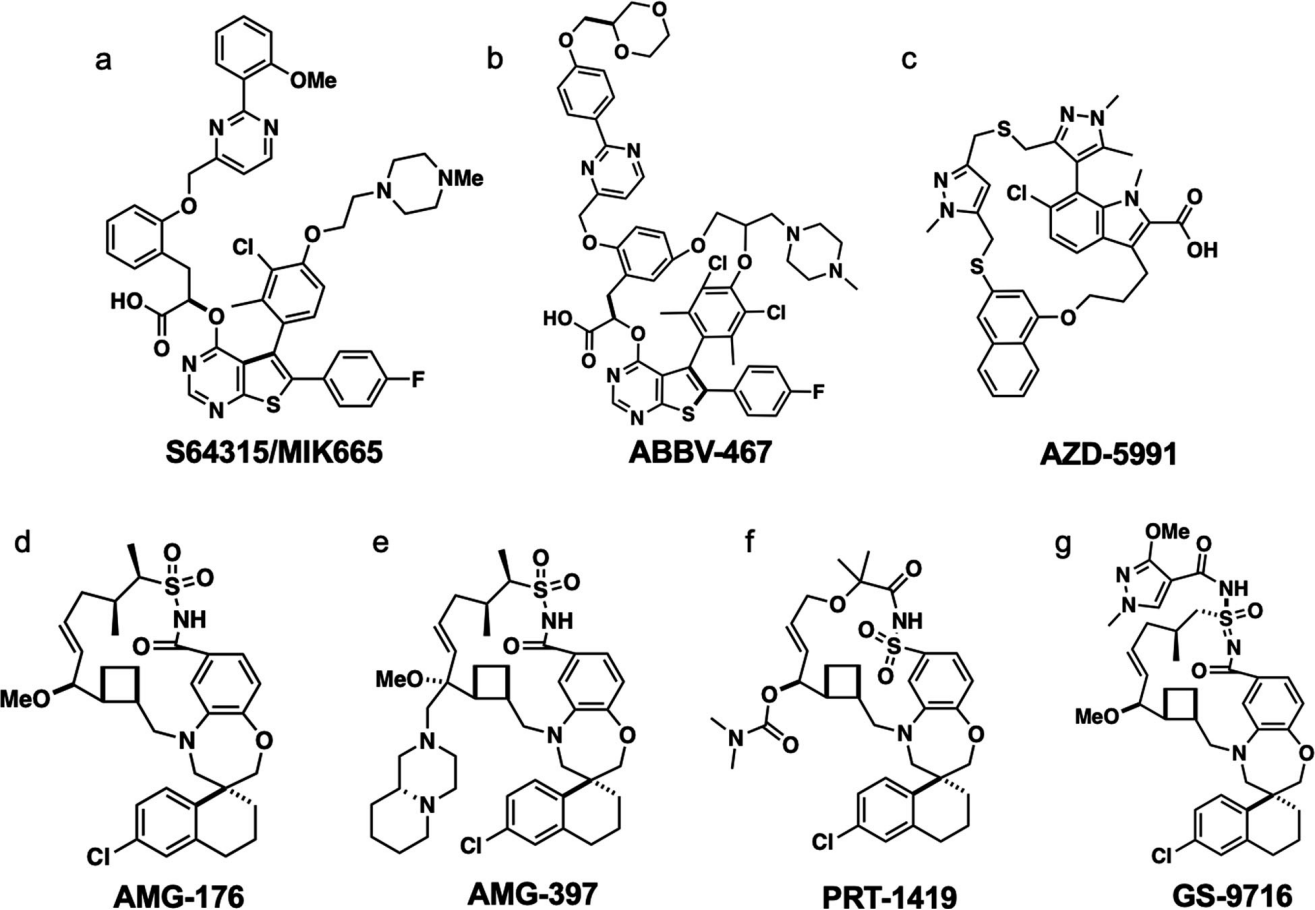
The structures of MCL-1 inhibitors that entered clinic trials. **a** S64315/MIK665, **b** ABBV-467, **c** AZD-5991, **d** AMG-176, **e** AMG-397, **f** PRT-1419, and **g** GS-9716 [136–138, 140, 142, 143].

Another antiapoptotic member of the BCL-2 family that has been targeted for the treatment of cancer is MCL-1 [133, 134]. Amplification of the MCL-1 gene is one of the most common genetic alterations observed in cancer, and MCL-1 is overexpressed in both hematologic malignancies and solid tumors [135]. However, like other members of this family, the discovery of a potent MCL-1 inhibitor was expected to be challenging due to the large protein-protein interface and the need for extremely tight binding (single digit picomolar). Nevertheless, several groups discovered very potent small molecule inhibitors of MCL-1 [136–143] and some have entered clinical trials. The MCL-1 inhibitors fall into three structural classes. Figure 8a, b depict MCL-1 inhibitors from Servier/Norvartis [140] and Abbvie [142], Fig. 8c depict an AstraZeneca MCL-1 inhibitor [138], and Fig. 8d–g depict MCL-1 inhibitors discovered by Amgen [136], Prelude [143], and Gilead [137]. Dose limiting cardiotoxicities were observed in the clinic for the MCL-1 inhibitors [142] despite the differences in their structures, suggesting that the observed cardiotoxicity is mechanism-based as postulated earlier by Joe Opferman [55, 68].

In summary, the antiapoptotic members of the BCL-2 family are excellent cancer targets and proved to be very druggable despite being involved in protein-protein interactions. However, in addition to killing cancer cells, some of the BCL-2 family inhibitors also kill normal cells making it difficult to achieve a therapeutic window limiting their utility. Attempts to overcome this limitation have recently been tried, including the use of antibody-drug conjugates [131], PROTACs that can selectively degrade a BCL-2 family protein in a tumor but not in a normal cell [144, 145] or by altering the physical properties of the inhibitor to restrict it from getting into the heart [142]. These strategies may produce BCL-2 family inhibitors/degraders in the future that will join Venetoclax as approved drugs to treat cancer.
